# Calibration of an Outdoor Distributed Camera Network with a 3D Point Cloud

**DOI:** 10.3390/s140813708

**Published:** 2014-07-29

**Authors:** Agustín Ortega, Manuel Silva, Ernesto H. Teniente, Ricardo Ferreira, Alexandre Bernardino, José Gaspar, Juan Andrade-Cetto

**Affiliations:** 1 Institut de Robòtica i Informàtica Industrial, CSIC-UPC, Llorens Artigas 4-6, Barcelona 08028, Spain; E-Mail: ehomar@iri.upc.edu; 2 Institute for Systems and Robotics, Instituto Superior Técnico, Av. Rovisco Pais 1, Lisbon 1049-001, Portugal; E-Mails: manuel.silva.thesis@gmail.com (M.S.); ricardo@isr.ist.utl.pt (R.F.); alex@isr.ist.utl.pt (A.B.); jag@isr.ist.utl.pt (J.G.)

**Keywords:** camera network calibration

## Abstract

Outdoor camera networks are becoming ubiquitous in critical urban areas of the largest cities around the world. Although current applications of camera networks are mostly tailored to video surveillance, recent research projects are exploiting their use to aid robotic systems in people-assisting tasks. Such systems require precise calibration of the internal and external parameters of the distributed camera network. Despite the fact that camera calibration has been an extensively studied topic, the development of practical methods for user-assisted calibration that minimize user intervention time and maximize precision still pose significant challenges. These camera systems have non-overlapping fields of view, are subject to environmental stress, and are likely to suffer frequent recalibration. In this paper, we propose the use of a 3D map covering the area to support the calibration process and develop an automated method that allows quick and precise calibration of a large camera network. We present two cases of study of the proposed calibration method: one is the calibration of the Barcelona Robot Lab camera network, which also includes direct mappings (homographies) between image coordinates and world points in the ground plane (walking areas) to support person and robot detection and localization algorithms. The second case consist of improving the GPS positioning of geo-tagged images taken with a mobile device in the Facultat de Matemàtiques i Estadística (FME) patio at the Universitat Politècnica de Catalunya (UPC).

## 1. Introduction

**Figure 1.**
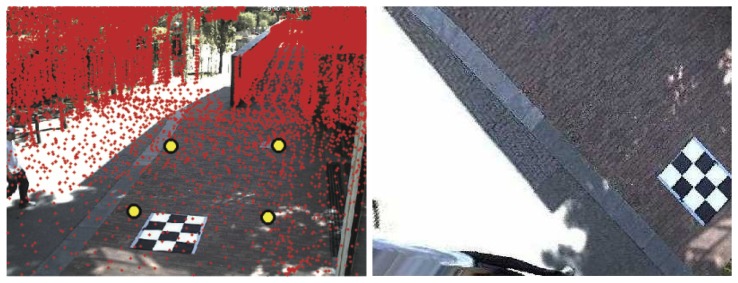
Results of the proposed calibration system. (**Left**) Plane selection in a graphical user interface and registration of the laser range data with a view from one of the cameras in the network; (**Right**) recovered orthographic view of the ground plane. The chess pattern shown is not used for calibration; it serves just to visually evaluate the quality of the ground-plane rectifying homography.

The development of powerful laser sensors combined with simultaneous location and mapping (SLAM) methodologies [1] allows the possibility to have available high precision 3D maps registered over large areas [2]. These maps come usually in the form of large point clouds and are typically used to support robot navigation systems [3]. These point clouds are usually acquired with laser range finders, cover the complete area of the network and, in particular, contain the areas corresponding to the fields of view of the cameras. This paper exploits these technologies, proposing a methodology to calibrate an outdoor, non-overlapped, distributed camera network using such range data. See Figure 1.

Traditional techniques for camera calibration require the use of non-planar [4] or planar [5] patterns, usually made of points, lines or checkerboards [6,7], conics [8] or, even, ARTagmarkers [9]. Unfortunately, for large outdoor camera networks, calibration patterns of reasonable sizes often project on images with very small resolution, mainly because cameras are usually located at a considerable height with respect to the floor, consequently making pattern segmentation difficult. In addition, a pattern-based independent calibration of each camera would require a secondary process to relate all camera coordinate systems to a global reference frame, but establishing this relation with small to null overlapping fields of view is nearly impossible. For planar scenarios, a direct linear transformation (DLT) [10] suffices to estimate image to plane homographies [11]. Unfortunately, the planar scenario assumption is too restrictive, especially in situations with nonparallel, locally planar surfaces, such as ramps and plazas, which often occur in real urban environments, as in our case.

An interesting technique to calibrate the camera network without the need of a pattern is with the aid of a bright moving spot [12]. The technique assumes overlapping fields of view to estimate the epipolar geometry to extract homographies, estimate depth and, finally, compute the overall calibration of the camera network. In our case, the cameras' fields of view seldom overlap, and the visibility of the bright spot does not always hold in sunlight. Another alternative is to place an LED light on a moving robot and to track it with a secondary robot equipped with a laser sensor, relating their position estimates to the camera network [13]. Yet another system that relies on tracking a moving object to estimate the extrinsic parameters is [14], which assumes a constant velocity model for the target. Tracking a moving target each time the system needs recalibration might be prohibitive. The estimation of the camera location purely by analyzing cast shadows is also mathematically possible, but with very low position accuracy in practice [15], and if one is interested only in the topology of the network configuration and not in a metric calibration, multi-target tracking of people could also be an alternative [16]. In contrast to these approaches, we opt for a system that does not rely explicitly on a moving pattern or shadow to calibrate the network and that produces an accurate metric calibration.

For camera network systems that incorporate camera orientation control (pan and tilt) and motorized zoom, it is possible to use such motion capabilities to first estimate the intrinsic parameters rotating and zooming, fitting parametric models to the optical flow, and then to estimate extrinsic parameters aligning landmarks to image features [17]. Unfortunately, in our case, the cameras are not active. Another option is to use stereo pairs instead of monocular cameras at each node in the network. In this way, local 3D reconstruction can be obtained directly within each node and registered globally using graph optimization techniques [18]. Overlapping fields of view are still necessary in that case. A third option to calibrate the camera network, albeit relative translation, is to use a vertical vanishing point and the knowledge of a line in a plane orthogonal to the vertical direction on each camera image [19]. A different, but related, problem is the relative positioning of one or more cameras with respect to a range sensor. To that end, calibration can be achieved using a checkerboard pattern, as in [20]. A related methodology to calibrate extrinsically an omnidirectional camera using point correspondences between a laser scan an the camera image is proposed in [21]. In contrast to our approach, the method assumes known intrinsic camera parameters. For a method to calibrate the laser intrinsic parameters instead, the reader is referred to [22].

We benefit from the availability of a dense point cloud acquired during a 3D laser-based SLAM session with our mapping mobile robot [3]. The set contains over eight million points and maps the environment with accuracies that vary from 5 cm to 20 cm, approximately. These data replace the need for a checkerboard pattern, a tracked beam, a robot or active capabilities of the cameras and are used as external information to calibrate the network.

Our work is largely related in spirit to the method described in [23], in which a set of images are registered into a urban point cloud. One major difference of the approach is on the assumptions made with respect to the characteristics of the scene during 3D feature extraction. In particular, the above-mentioned technique exploits the fact that buildings have strong parallel edges and that these cluster with similar orientation. On the contrary, we exploit the fact that in urban scenes, large planar regions also meet at long straight edges. In contrast with [24], in which edge parallelism is used to calibrate only the attitude and focal length of cameras for traffic monitoring, we use edge information to calibrate also the camera location.

The rest of the paper is organized as follows. We explain first how nominal calibration is executed and then how this calibration is refined, first by extracting 3D features from the point clouds and optimizing over the reprojection error of their matching to those found in images. When needed, a final refinement step is computed by means of the DLT-lines algorithm. Experiments on a pair of scenarios are presented to show the feasibility of the proposed solution. The paper is concluded with some remarks and future work.

## 2. Nominal Calibration

**Figure 2.**
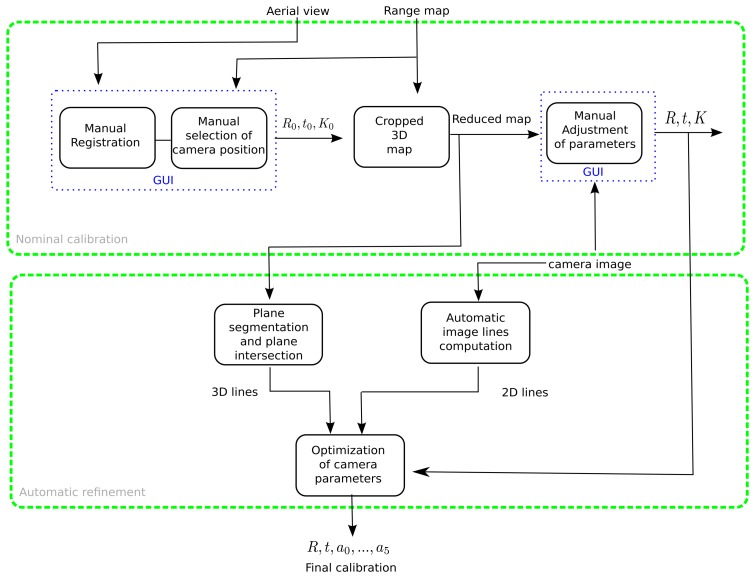
Two-step calibration methodology. In the first step, a graphical user interface is used to assist in an initial manual registration of the point cloud. The second step refines this registration, matching 2D image lines with 3D edges in the point cloud.

The proposed calibration procedure is illustrated in Figure 2. It consists of two main steps. In the first step, the internal camera parameters are initialized to the manufacturer specs (image pixel width and depth and focal length), and a nominal calibration of the camera external parameters is obtained by manually registering the point cloud to an aerial image of the experimental site with the aid of a graphical user interface, prompting the user to coarsely specify the camera location, orientation, height and field of view. These initial parameters allow the cropping of the full point cloud into smaller regions of interest compatible with the field of view of each camera. The user can then adjust the registration by manually modifying each of the parameters (see the video associated with [25], available in the IEEE Xplore digital library).

In the second step, an automatic refinement of the camera calibration parameters is obtained by matching 2D image lines to their corresponding 3D edges in the point cloud. To this end, the point cloud is segmented into a set of best fitting planes with large support using local variation [26], and straight edges are computed from the intersection of perpendicular planes in the set. The extracted 3D lines are associated with 2D image lines, and this information is fed to a non-linear optimization procedure that improves both intrinsic and extrinsic parameters. Finally, the homographies of the walking areas are computed for the planar regions in the range data. The final output of the whole calibration procedure consists in: (1) the extrinsic camera parameters, *i.e.*, the position and orientation of each camera in the world frame; (2) the intrinsic camera parameters (focal distance, image center aspect ratio and distortion terms); and (3) the homographies of each walking area.

The first step of the calibration procedure needs to be performed only once, during the camera network installation or when the network topology changes, *i.e.*, cameras are added/moved, and takes only a couple of minutes. The second step, which does not require user intervention, can be executed as frequently as needed to keep the system calibrated, despite small modifications in camera orientation due to weather conditions and maintenance operations. In the following paragraphs, we detail the nominal calibration.

For the nominal calibration, the user interacts with a graphical user interface to coarsely locate each camera with respect to the reference frame of the point cloud and the viewing direction in the ground plane. For an easier interaction, the point cloud appears registered with an aerial view of the environment. The registration of the point cloud with the aerial view is computed manually using the DLT [10]. The camera parameters are initialized with default intrinsic parameters.

**Figure 3.**
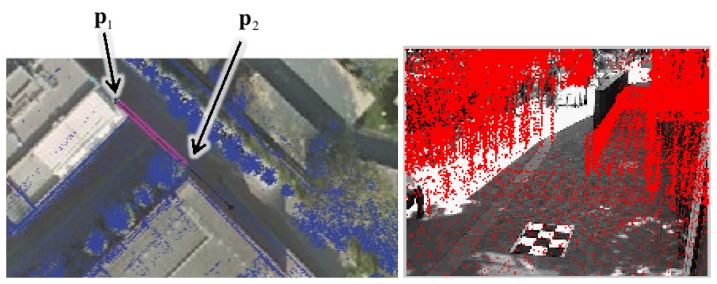
Graphical user interface. (**Left**) The point cloud is shown to the user overlaid on top of an aerial view of the environment. The user is prompted to select (1) a coarse camera location **p**_1_; and (2) the viewing direction **p**_2_ indicated by the magenta line; (**Right**) During the initialization process, the user can manually adjust intrinsic and extrinsic parameters on a projected view of the point cloud.

We then compute initial values for the camera pose: the center of projection **t** is simply the user selected point **p**_1_, located at a user define height (*i.e.*, 6 m in our application). See Figure 3. The two selected points **p**_1_ and **p**_2_ set the azimuth direction *ψ*. The elevation φ gives a user-defined inclination to the ground (17° in the shown example), and the roll *ρ* is set to *π* to account for the proper axes changes. These parameters suffice to compute the initial rotation matrix **R** = **R***_ρ_***R***_ψ_***R***_φ_* with:

$${\text{R}}_{\psi }=\left[\begin{array}{ccc} \cos(\psi ) & \sin(\psi ) & 0\\ -\sin(\psi ) & \cos(\psi ) & 0\\ 0 & 0 & 1 \end{array}\right],{\text{R}}_{\phi }=\left[\begin{array}{ccc} \cos(\phi ) & 0 & -\sin(\phi )\\ 0 & 1 & 0\\ \sin(\phi ) & 0 & \cos(\phi ) \end{array}\right],\text{and}\;{\text{R}}_{\rho }=\left[\begin{array}{ccc} 0 & -1 & 0\\ 0 & 0 & -1\\ 1 & 0 & 0 \end{array}\right]$$

Assuming that the principal point (*u*_0_, *v*_0_) is located at the image center, we can compute an initial estimate for the camera intrinsic parameters using as input the focal length *f* and the CCD pixel size in millimeters *k_u_* and *k_v_*, *i.e.*, *α_v_* = *f k_v_*, *α_u_* = *f k_u_*, and:

(1) $$\text{K}=\left[\begin{array}{ccc} a_{u} & 0 & u_{0}\\ 0 & \alpha_{v} & v_{0}\\ 0 & 0 & 1 \end{array}\right]$$

The initial estimate of the perspective projection matrix for each camera is:

(2) P=K[R|t]

Once this initial estimate is obtained for a particular camera, the user can further adjust each parameter manually, whilst a projection of a cropped section of the point cloud that falls within the viewing frustum is visualized in the image. Note, however, that this initial estimate does not take into account radial distortion parameters. These are refined along with the rest of parameters in the second step of the method.

## 3. Calibration Refinement

To improve camera calibration from the nominal parameters, we propose an automatic method, where relevant 3D edges are extracted from the point cloud and matched to corresponding image lines. In practice, the method works well with about a half-dozen lines selected from each camera image.

The procedure uses the nominal calibration as an initial rough approximation and can be executed anytime to recover from small perturbations in camera orientation or any other parameter, due to weather (wind, rain, *etc.*) or maintenance operations (repair, cleaning).

### 3.1. 3D Edge Computation

**Figure 4.**
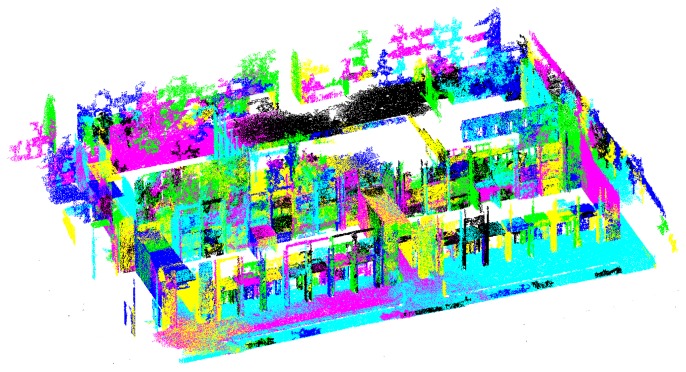
Segmentation of a point cloud into planar regions. Colors represent individual planar regions detected by the algorithm.

The computation of straight lines from the point cloud relies on identifying and intersecting planes. The method to segment planar regions is motivated by Felzenszwalb's algorithm to 2D image segmentation [27] and extended to deal with non-uniformly sampled 3D range data [26]. The algorithm sorts point to point distances for each point's k-nearest neighbors and then traverses the list of sorted distances in increasing order, growing planar patches by joining points that meet two matching criteria, *i.e.*, distance constraints and orientation constraints. Thanks to the use of union by rank and path compression [28], the algorithm runs nearly in linear time with respect to the number of points in the point cloud. The ANN library proved an adequate tool for the computation of approximate nearest neighbors [29]. If computation time becomes an issue, libnabo could be used as an alternative [30]. Figure 4 shows an example of the segmentation results. In the figure, all points corresponding to the same planar patch are represented with the same color. Note, however, that even when colors repeat for different planes, their labels are different.

In the segmentation algorithm, local surface normals **n** are computed for each point in the point cloud, fitting local planar patches. To account for global variation, planar patches are merged, growing a forest of trees based on curvature and mean distance. The curvature between two candidate regions is computed from the angle between their two normals, which must be below a user-selected threshold *t_c_*,

(3) $$|\arccos({\text{n}}_{i}^{T}{\text{n}}_{j})|\;<t_{c}$$

**Figure 5.**
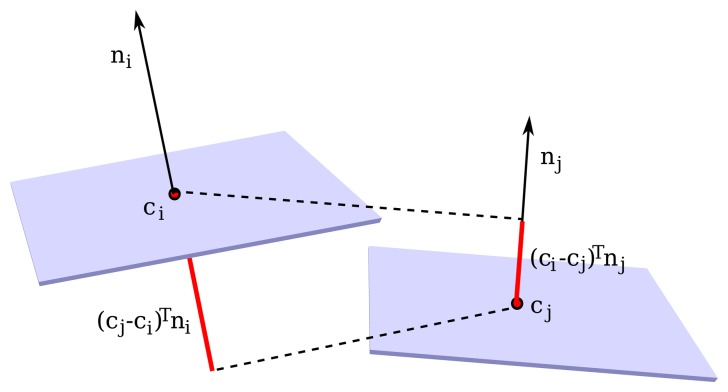
Projection of the region centers **c***_i_* and **c***_j_* onto neighboring planar patches.

Two segments passing the curvature criteria are merged if they also pass a distance constraint. That is, if the sum of distances between their centers along weighted orthogonal directions is below a user-selected threshold *t_d_*,

(4) $$\frac{k_{i}\left|{\left({\text{c}}_{i}-{\text{c}}_{j}\right)}^{T}{\text{n}}_{j}\right|+k_{j}\left|{\left({\text{c}}_{j}-{\text{c}}_{i}\right)}^{T}{\text{n}}_{i}\right|}{k_{i}+k_{j}}<t_{d}$$

with *k_i_* and *k_j_* the number of points each segment holds and **c***_i_* and **c***_j_* the segment centers. See Figure 5.

Once a set of segments is obtained, their intersecting edges are computed, and the ones with sufficient support from their generating planes and with good orthogonality conditions are selected for projection onto the images. The method is summarized in Algorithm 1.


**Algorithm 1** The algorithm to find edge lines at orthogonal plane intersections within the point cloud.
EdgeExtraction(*M*)Input: *M* : Point cloud.Output: *E*: 3D lines.1:*D* ← ø2:**for** each **p***_i_* ∈ *M*
**do**3: *N_i_* ← FindNeighbors(**p***_i_, M*)4: **n***_i_* ← ComputeNormal(**p***_i_, N_i_*)5: Label(**p***_i_*) ← *i*6: *D* ← *D* ⋃ FindDistances(**p***_i_, N_i_*)7:**end for**8:*D* ← SortDistances(*D*)9:**for** each *d_k_* ∈ *D*
**do**10: **c***_i_* ← Start(*d_k_*)11: **c***_j_* ← End(*d_k_*)12: **if** Label(**c***_i_*) ≠ Label(**c***_j_*) **then**13:  **if**
$|\cos^{-1}({\text{n}}_{i}^{T}{\text{n}}_{j})|<t_{c}$
**then**14:    **if**
$\frac{k_{i}|{\left(c_{i}-c_{j}\right)}^{T}{\text{n}}_{j}|+k_{j}|{\left({\text{c}}_{j}-{\text{c}}_{i}\right)}^{T}{\text{n}}_{i}|}{k_{i}+k_{j}}<t_{d}$
**then**15:    MergeTrees(**c***_i_*, **c***_j_*)16:   **end if**17:  **end if**18: **end if**19:**end for**20:*E* ← ø21:*L* ← Labels(*M*)22:**for** each pair of segments (**s***_i_*, **s***_j_*) ∈ *L* with respective (**n***_i_*, **n***_j_*) **do**23: **if** Orthogonal(**n***_i_*, **n***_j_*) **then**24:  *E* ← *E* ⋃ PlaneIntersection(**n***_i_*, **n***_j_*)25: **end if**26:**end for**


### 3.2. Optimization

Straight image lines are extracted from the camera images using the method proposed in [31]. The line set is pruned to those lines larger than a predefined threshold.

3D model lines are projected onto the image plane using the projection matrix computed during the nominal calibration step. 2D-3D line association is attained by matching such projections to the closest 2D image line.

**Figure 6.**
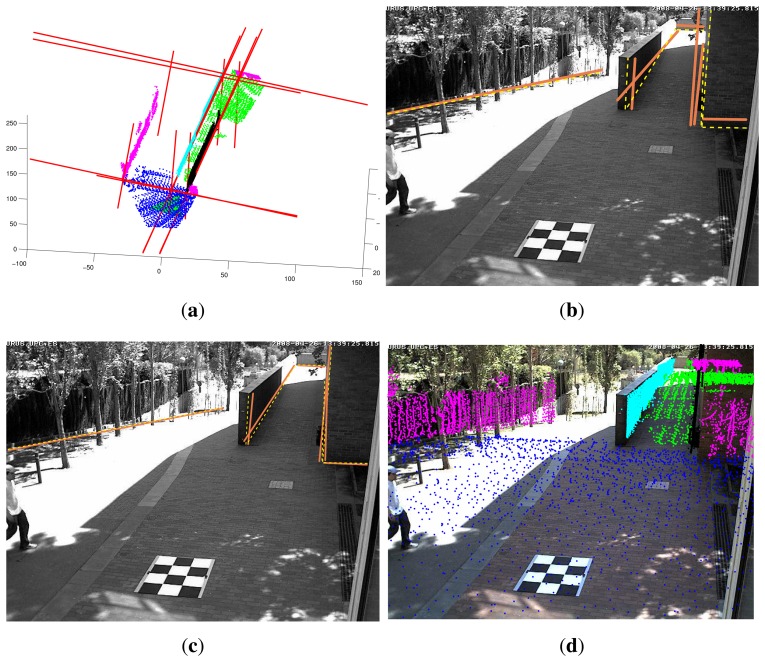
Optimization. (**a**) Computation of plane intersections in the range data; (b) Projection of lines onto the image plane using the nominal calibration parameters; (c) line matching optimized in the image plane; (**d**) segmented point cloud reprojected onto the calibrated image.

Once the 3D-2D association is established, nonlinear optimization is performed to improve the nominal calibration by minimizing the squared sum of the line endpoint projection errors:

(5) $$\underset{\vartheta }{\text{minimize}}{\sum_{i}\left\|{\text{u}}_{i}-{\text{u}}_{id}(\vartheta )\right\|}^{2}$$

where **u***_id_*(ϑ) is the distorted projection of the 3D endpoint **p***_i_*, ϑ = (**K****, **R****, **t**, *a*_1_, *a*_2_) are the set of parameters being optimized and **u***_i_* is the image point. The optimization is solved using Levenberg–Marquardt nonlinear optimization. See Figure 6.

In this step of the method, image distortion is modeled based on even powers of the radial distance in the image plane:

(6) $${\text{u}}_{id}={\text{u}}_{in}+(1+\sum_{j=1}^{2}a_{j}r^{2j})({\text{u}}_{in}-{\text{u}}_{0})$$

where *a_j_* are the distortion parameters, *r*^2^ = ‖**u***_in_* − **u**_0_‖^2^ is the radial distortion factorization, **u**_0_ is the computed principal point, **u***_in_* is the normalized (pinhole) image projection of point **p***_i_*:

(7) $$\left[\begin{array}{l} {\text{u}}_{in}\\ 1 \end{array}\right]\sim \text{K}[\text{R}|\text{t}]\left[\begin{array}{l} {\text{p}}_{i}\\ 1 \end{array}\right]$$

and ∼ denotes equality up to a scale factor.

#### 3.2.1. Initialization of the Optimization

The nominal calibration introduced in Section 2 is, in general, sufficient to initialize the calibration optimization formalized in Equation (5). However, one finds that while it is usually simple and very effective to observe precisely some parameters as the camera horizontal position in the coordinates of a laser range finder map, other parameters, such as camera height, 3D rotation, focal length or principal point, are more challenging. The possibility of automating the initialization of the optimization procedure is a convenient feature for calibrating cameras in a network.

The initialization of the calibration optimization process may be setup to find from zero till all of the calibration parameters. Zero means using all of the nominal calibration results for initializing the optimization. All means (re-)finding all initialization parameters from image and laser range finder data. In between are several cases of interest, many of which have solutions published. For example, if a camera has its intrinsic parameters calibrated before being mounted in place, then one just has to estimate the extrinsic parameters by solving the well-known Perspective-n-Point (PnP) problem [32]. In the following, we detail two cases. In the first case, we show how to estimate all of the parameters from 3D lines imaged by the camera to calibrate. In the second case, we consider that the camera position is precisely known and detail how to estimate the intrinsic parameters and 3D rotation.

As proposed in [33], the use of image lines instead of isolated points in the camera calibration process brings an advantage. Image processing can be used for fine tuning the location of the lines in the image and therefore automatically improving the calibration data input. In this section, *DLT-Lines* is presented as a method to initialize the optimization step, allowing one to estimate simultaneously the camera projection matrix and radial distortion, from the 3D point cloud and 2D lines.

Considering the shorthand notation for image points ${\text{m}}_{i}={\left[{\text{u}}_{i}^{T}\;1\right]}^{T}$ and 3D points ${\text{M}}_{i}={\left[{\text{p}}_{i}^{T}\;1\right]}^{T}$ the perspective camera model, Equation (7), becomes **m***_i_* ~ **PM***_i_*.

The projection of a 3D line **L***_i_* to the camera image plane can be represented by the cross product of two image points in projective coordinates:

(8) $${\text{l}}_{i}={\text{m}}_{1i}\times {\text{m}}_{2i}$$

Any point **m***_ki_* lying in the line **l***_i_* implies that ${\text{l}}_{i}^{T}\;{\text{m}}_{ki}=0$. Hence, applying the multiplication of ${\text{l}}_{i}^{T}$ to both sides of the perspective camera model, *i.e.*, ${\text{l}}_{i}^{T}\;{\text{m}}_{ki}={\text{l}}_{i}^{T}\text{P}\;{\text{M}}_{ki}$ leads to:

(9) $${\text{l}}_{i}^{T}\;\text{P}\;{\text{M}}_{ki}=0$$

where **M***_ki_* is a 3D point in projective coordinates lying in **L***_i_*. The properties of the Kronecker product [34] allow one to obtain a form factorizing the vectorized projection matrix:

(10) $$\left({\text{M}}_{ki}^{T}⊗{\text{l}}_{i}^{T}\right)\text{vec}(\text{P})=0$$

Considering *N* ≥ 12 pairs (**M***_ki_*, **l***_i_*), one forms a matrix **B**, *N* × 12, by stacking the *N* matrices ${\text{M}}_{ki}^{T}⊗{\text{l}}_{i}^{T}$. An example of *N* = 12 arises when one observes six 2D lines imaging six 3D lines, *L_i_* (*i* = 1, …, 6), each one represented by two end points, *L_i_* ↔ (*M_i_*_1_ *M_i_*_2_). Alternatively, given a 3D line **L***_i_* and its projection represented by the image line **l***_i_*, any 3D point lying on the 3D line **L***_i_* can be paired with the 2D line **l***_i_*. On the other hand, any image line **l***_i_* can be paired with any 3D point lying on **L***_i_*, *i.e.*, more than one image line can be paired with a 3D point.

The least squares solution, more precisely the minimizer of ‖**B** *vec*(**P**)‖^2^ subjected to ‖*vec*(**P**)‖ = 1, is the right singular vector corresponding to the least singular value of **B**.

Note that the perspective camera model, as presented in Equation (7), does not contain yet the radial distortion. To include radial distortion, we use Fitzgibbon's division model [35]. An undistorted image point, **u** = [*u v*]*^T^*, is computed from a radially distorted image point **u***_d_* = [*u_d_*   *v_d_*]*^T^* as **u** = **u***_d_/*(1 + *λ* ‖**u***_d_*‖^2^), where *λ* represents the radial distortion parameter. The division model allows one to define a line **l**_12_ as the cross product of two points:

(11) $${\text{l}}_{12}=\left[\begin{array}{c} u_{1d}\\ v_{1d}\\ 1+\lambda s_{1}^{2} \end{array}\right]\times \left[\begin{array}{c} u_{2d}\\ v_{2d}\\ 1+\lambda s_{2}^{2} \end{array}\right]={\hat{\text{l}}}_{12}+\lambda {\text{e}}_{12}$$

where *s_i_* is the norm of distorted image point *i*, $s_{i}^{2}=u_{id}^{2}+v_{id}^{2}$, the distorted image line is denoted as ${\hat{\text{I}}}_{12}={\left[u_{1d}v_{1d}1\right]}^{T}\times {\left[u_{2d}v_{2d}1\right]}^{T}$ and the distortion correction term $e_{12}={\left[v_{1d}s_{2}^{2}-v_{2d}s_{1}^{2},u_{2d}s_{2}^{2}-u_{1d}s_{2}^{2},0\right]}^{T}$.

Applying Equation (11) on Equation (10) leads to the following equation:

(12) $$\left({\text{M}}_{k12}^{T}⊗{({\hat{\text{I}}}_{12}+{\lambda \text{e}}_{12})}^{T}\right)\;\text{vec}(\text{P})=0$$

which can be rewritten as:

(13) $$\left({\text{B}}_{ki1}+\lambda {\text{B}}_{ki2}\right)\text{vec}\left(\text{P}\right)=0$$

where ${\text{B}}_{ki1}={\text{M}}_{k_{12}}^{T}⊗{\hat{\text{l}}}_{12}^{T}$, ${\text{B}}_{ki2}={\text{M}}_{k12}^{T}⊗{\text{e}}_{12}^{T}$ and **M***_k_*_12_ denotes the *k* − *th* 3D point projecting to the distorted line **Î**_12_.

To solve Equation (13) instead of Equation (10), we still need to consider *N* ≥ 12 pairs (**M***_ki_*, **Î***_i_*), where *N* = *k_max_i_max_*, and form now two *N* × 12 matrices, **B**_1_ and **B**_2_, instead of just **N**, by stacking matrices **B***_ki_*_1_ and **B***_ki_*_2_. Left-multiplying the stacked matrices by ${\text{B}}_{1}^{T}$ results in a polynomial eigenvalue problem (PEP), which can be solved, for example, in MATLAB using the polyeig function. It gives, simultaneously, the projection matrix, in the form of *vec*(**P**), and the radial distortion parameter *λ*.

Having estimated the projection matrix, **P**, the camera intrinsic and extrinsic parameters can be obtained by QR-decomposition [10]. More precisely, given the sub-matrix **P**_3×3_ containing the first three columns of **P** and **S** an anti-diagonal matrix:

(14) $$\text{S}=\left[\begin{array}{ccc} 0 & 0 & 1\\ 0 & 1 & 0\\ 1 & 0 & 0 \end{array}\right]$$

the QR-decomposition allows factorizing **P**_3×3_*^T^* **S** = **QU**, where **Q** is an orthogonal matrix and **U** is an upper triangular matrix. Then, the intrinsic parameters and the rotation matrices are computed as **K** = −**SU***^T^* **S** and **R** = **Q***^T^* **S**. Finally, the camera position is obtained with **t** = **KP**_4_, where **P**_4_ is a 3 × 1 vector containing the fourth column of **P**. If the diagonal of **K** contains negative values, then it is corrected by post-multiplying by a diagonal matrix. In MATLAB/Octave D= diag(sign(diag(K))); K= K*D; R= D*R; t= D*t;. In addition, since ±**P** are both solutions of Equation (10), the factorization of **P** may imply *det*(**R**) = −1. If *det*(**R**) = −1, then the factorization of **P** is repeated using −**P**.

To convert the obtained distortion parameter *λ* to the distortion parameters in Equation (6), we sample the region of interest and find a least squares solution for the best parameter fit in this region. Starting from a set of camera points evenly sampled at pixel granularity covering the image dimensions {**u***_id_*}, we apply the Fitzgibbon distortion model to obtain an uneven set of undistorted pixel coordinates {**u***_i_*}. We next solve the optimization problem:

(15) $$\underset{a_{j}}{\text{minimize}}{\sum_{i}\left\|{\text{u}}_{0}+\left(1+\sum_{j=1}^{2}a_{j}r^{2j}\right)({\text{u}}_{i}-{\text{u}}_{0})-{\text{u}}_{id}\right\|}^{2}$$

which is a linear least squares problem in the variables *a_j_*, where a closed form solution is available. For small distortions, we empirically find that *a*_1_ = *λ* and *a*_2_ = 0 provide a good fit to initialize the main optimization algorithm.

#### 3.2.2. Known Camera Location

In the case one knows accurately the camera location, e.g., the camera has been imaged by the 3D data acquisition system, then the number of degrees of freedom of the calibration problem is decreased. The DLT methodology presented in the previous section can be further simplified.

Subtracting the camera center to all points of the point cloud results in a coordinate system where the camera is at the origin, and thus, the projection matrix, **P** = **K**[**R**| **t**] is equivalent to a simple homography, $\hat{\text{P}}=\text{K}R$. Considering image lines **l***_i_* and 3D points, **p***_ki_* = [*x_ki_ y_ki_ z_ki_*]*^T^*, imaged as points of the lines, recalling Equation (9), one has:

(16) liTKR(pki−tWC)=0

where *^W^***t***_C_* denotes the camera projection center in world coordinates. As such, one obtains linear constraints similar to the ones already derived for *DLT-Lines*:

(17) ((pki−tWC)T⊗liT)vec(KR)=0

The length of *vec*(**KR**) is just nine, *i.e.*, the knowledge of the camera location saves three variables to estimate, and thus, the estimation process is intrinsically simplified. Finally, the projection matrix, **P**, can be obtained decomposing $\hat{\text{P}}=\text{K}R$ and adding the camera location as $\text{P}=\left[\hat{\text{P}}|{\hat{\text{P}}}^{w}{\text{t}}_{C}\right]$.

The calibration problem has been reduced to the estimation of a homography, represented by *vec*(**KR**) and, therefore, not including radial distortion. A similar form based on Equation (17) can be obtained for the radial distortion case represented in Equation (12):

(18) $$\left({\tilde{\text{p}}}_{k12}^{T}⊗{({\hat{\text{l}}}_{12}+\lambda {\text{e}}_{12})}^{T}\right)\;vec(\text{KR})=0$$

where p∼;k12=(pk12−tWC). Equation (18) can be re-written in the form of Equation (13), which can be used to estimate the camera projection matrix **P** and radial distortion parameter *λ*, as shown before.

## 4. Experiments

**Figure 7.**
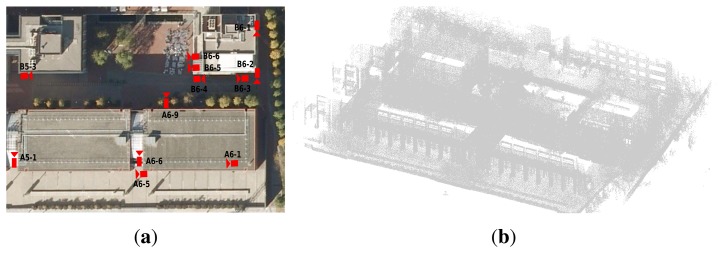
The Barcelona Robot Lab. (**a**) Aerial view of the camera distribution; and (**b**) the point cloud.

To show the cogency of the proposed calibration method, we show calibration results of two different outdoor scenarios. In both cases, the range data was gathered using a custom-built 3D laser with a Hokuyo UTM-30LX scanner mounted in a slip-ring. Each scan has 194,580 points with a resolution of 0.5° azimuth and 0.25° elevation. The first dataset was acquired in the Barcelona Robot Lab (BRL), located at the Campus Nord of the Universitat Politècnica de Catalunya (UPC). The BRL is a 10,000 m^2^ facility for research in outdoor service mobile robotics. It is equipped with a camera network, from which we only use 12 of the 21 cameras. They are shown in Figure 7. For this dataset, our 3D laser scanner was mounted on Helena, a Pioneer 3AT mobile robot, acquiring a total of 400 scans; however, only 30 of them were necessary to cover the area of the selected cameras. The complete dataset is available online [36].

**Figure 8.**
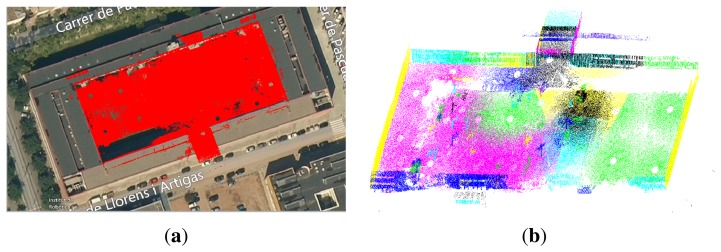
The Facultat de Matemàtiques i Estadística (FME) scenario. (**a**) The point cloud registered onto an aerial view of the scene; and (**b**) the segmented point cloud.

The second dataset was gathered in the inner courtyard of the Facultat de Matemàtiques i Estadística (FME), located at the Campus Sud of the UPC. For this dataset, the range sensor was mounted atop our robot Teo, a rough outdoor terrain Segway RPM400 mobile robot. In this case, only 39 scans were collected. Figure 8 shows the point cloud registered onto an aerial view of the scene and the segmented planes. A mobile phone camera was used to acquire geo-tagged images from different position in this scenario.

In both cases, the point clouds generated from the aggregation of multiple scans are preprocessed to remove outliers, to smooth out the planar regions and to provide a uniform point distribution through subsampling. The details of the filtering scheme used follow [3]. The parameters used to segment the range data in both scenarios were *n* = 25 neighbors to fit planar patches, a distance threshold of *t_d_* = 0.5 and a curvature threshold *t_c_* = 0.8. Furthermore, we only consider lines intersecting orthogonal planes with a deviation of ±3° from orthogonality. For those cases when there were less than six independent lines detected for the calibration of a camera, each detected line was broken into three segments, and all of these were used for the optimization step. This allowed one to have a sufficiently large number of lines to find a least squares solution to Equations (17) and (18). In most of those cases, however, the DLT-lines algorithm did not contribute to the improvement of the solution, and the first optimization sufficed to find acceptable results.

For the BRL dataset, the initial elevation angle was set to 17°. We initialized on this value, because most of the cameras are located about 6 m above the ground, and objects in the images for which lines can be detected reliably are closer than 20 m. The horizontal field of view is initialized to 40°, which corresponds to an 8-mm lens in a 0.25-in CCD.

**Figure 9.**
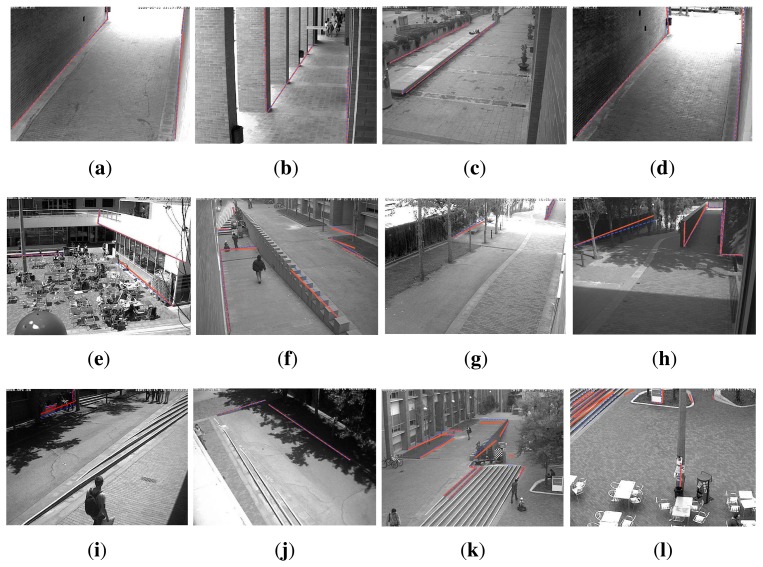
Results of the final calibration of the camera network for the BRL scenario. Optimization approximates the projected laser lines (**blue**) to the image lines (**red**). (**a**) A5-1; (**b**) A6-1; (**c**) A6-5; (**d**) A6-6; (**e**) A6-9; (**f**) B5-3; (**g**) B6-1; (**h**) B6-2; (**i**) B6-3; (**j**) B6-4; (**k**) B6-5; (**l**) B6-6.

The final calibration of the internal camera parameters for the BRL set are given in Table 1, along with the mean reprojection error and standard deviation. Note that a comparison of these estimates to those obtained with a checkerboard is unrealistic due to the actual positioning of the cameras. An unfeasibly large checkerboard would be needed and moved along the whole camera workspace to achieve significant results. Table 2 gives the obtained camera locations for this experiment, together with the camera ground truth locations manually extracted from the 3D point cloud. These values should only be used as a reference, since small variations of focal length might have incidence in the final positioning of the camera along the principal axis, without detriment to the camera reprojection for a limited range of depth values. The elapsed computation time for the calibration of each camera is also reported in the table. Figure 9 shows the reprojection of each matched line onto the corresponding camera images after the optimization is computed.

**Figure 10.**
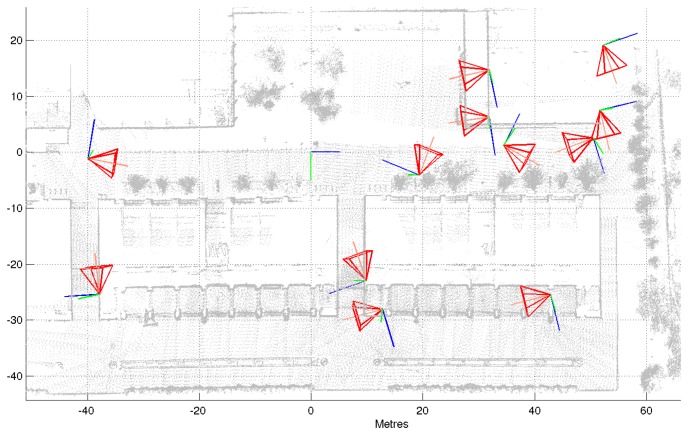
Calibrated camera locations for the Barcelona Robot Lab (BRL) dataset.

**Figure 11.**
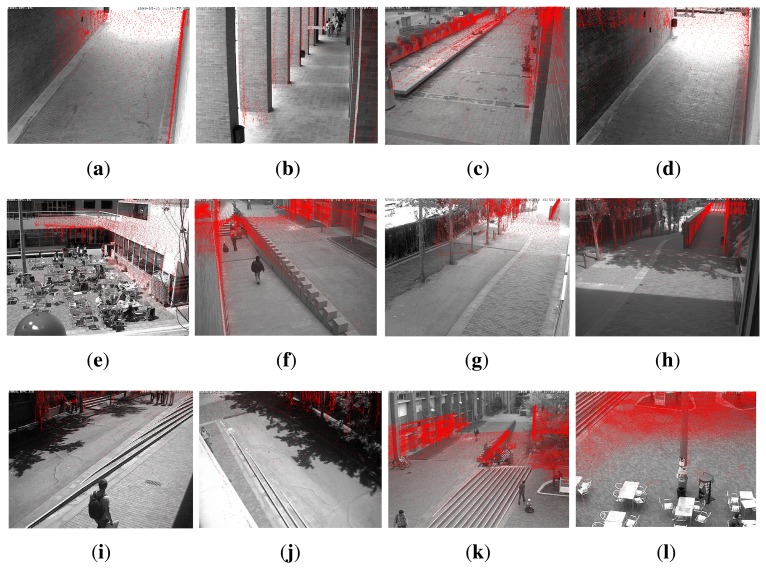
Visualization of range data on each of the camera views of the BRL scenario. (**a**) A5-1; (**b**) A6-1; (**c**) A6-5; (**d**) A6-6; (**e**) A6-9; (**f**) B5-3; (**g**) B6-1; (**h**) B6-2; (**i**) B6-3; (**j**) B6-4; (**k**) B6-5; (**l**) B6-6.

The estimated camera poses are plotted in Figure 10. Camera viewpoints are represented with triangular pyramids that suggest the viewing direction. To empirically judge the quality of the calibration results, we can also project all points from the map that fall in each camera viewing frustum onto the image plane. This is shown in Figure 11 for the BRL.

In the case of the FME scenario, all images were taken with a mobile phone. GPS readings on the phone were used as initial position estimates. The local world model was considered planar, so that a homography can be used to translate from GPS coordinates to the metric representation used in the point cloud.

Table 3 shows the results of the calibration of internal camera parameters. Since all images were computed using the same camera, the mean values obtained for the internal parameters can be used as a reference. These mean values are shown in the first line in the table.

Table 4 reports the different camera positions estimated with our algorithm and contrasted to the GPS readings on the phone. GPS coordinates are transformed to metric coordinates with the WGS84 standard and affine transformed with the DLT algorithm to align them with the FME building. Height values are not reported, as their readings from the phone GPS unit are unreliable.

**Figure 12.**
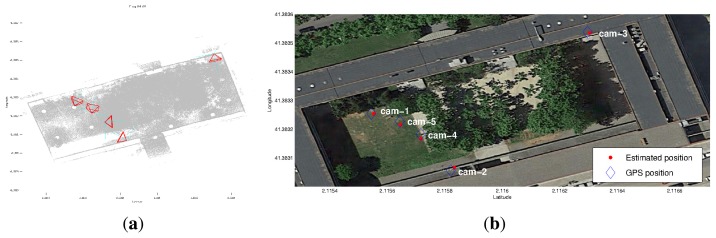
Camera localization for the FME scenario. (**a**) Camera viewpoint estimates; and (**b**) a comparison between GPS measures (blue squares) and our method (red points).

This comparison is shown schematically in Figure 12. Figure 12a shows the estimated camera viewpoints, whereas Figure 12b shows a comparison between GPS readings (blue squares) and the camera poses computed with our method (red dots).

**Figure 13.**
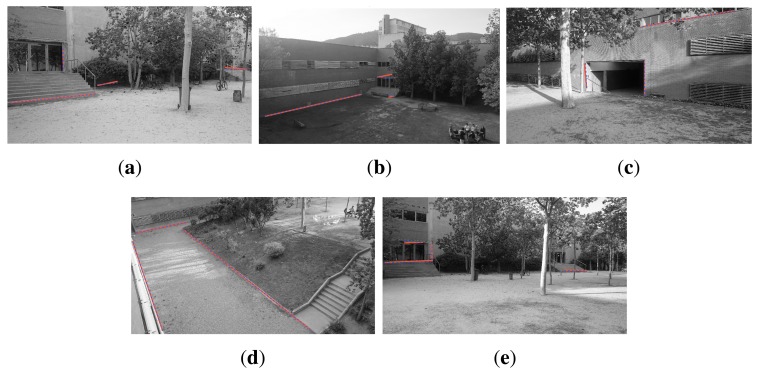
The results of the final calibration camera network. Optimization approximates the projected laser lines (blue) to the image lines (red) using the FME dataset. (**a**) Cam-1; (**b**) Cam-2; (**c**) Cam-3; (**d**) Cam-4; (**e**) Cam-5.

Figure 13 shows results of the optimization results for the FME dataset. In blue projected 3D lines and in red selected image lines. The heavy presence of unstructured data made it difficult to find a large number of support lines for calibration in this scenario, suggesting the need for calibration also with point/appearance features, together with lines. We leave this hybrid scheme as an open alternative for further development of the method.

### Computing Homographies

**Figure 14.**
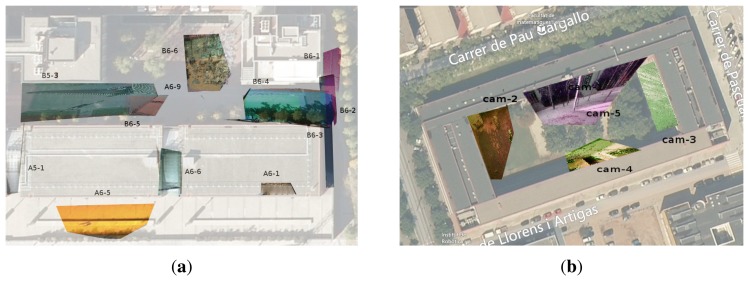
Computed homographies for the two scenarios. (**a**) BRL scenario; (**b**) FME scenario.

To measure events occurring on the scene, such as path lengths or areas of crowdedness, it would be necessary to obtain direct mappings from images to planar regions in the floor. The idea is to have a practical way to transfer 2D images to the world coordinates of the targets detected. To this end, we compute the homographies of user-selected planes with the end of a graphical user interface. The user selects polygonal regions in the images, and the 3D points that project inside these polygons are used to approximate the 3D planes. The algorithm to compute the homographies is the standard direct linear transform [10]. Figure 14 shows the result of this computation for the two scenarios. Camera images, each one of a different color mask, are projected onto their corresponding planar regions in the map.

## 5. Conclusions

In this paper, we have proposed a methodology to calibrate outdoor distributed camera networks having small or inexistent overlapping fields of view between the cameras. The methodology is based on the matching of line image features with 3D lines computed from dense 3D point clouds of the scene.

In the first stage, the user obtains the nominal calibration by using default intrinsic parameters for the cameras and indicating their positions and orientations on an aerial view aligned with the range map. Next, the calibration of each camera is improved by an automatic optimization procedure detecting lines in the 3D map and matching them with image lines. The lines are detected in the point cloud by automatically segmenting out planar regions and finding such plane intersections. The optimization procedure then minimizes the distance between points in the lines found in the map and their corresponding points in the image lines. The method has been used to calibrate the Barcelona Robot Lab, a 10,000 m^2^ area for mobile robot experimentation, and for camera localization at the FME patio, both located at the UPC campus in Barcelona.

Future work will include an analysis of uncertainty for the external calibration using the DLT-lines algorithm and the combination of feature points together with lines for scenes with a limited structure.

## Figures and Tables

**Table 1.** Estimated internal camera parameters of the BRL scenario.

**Camera**	**Focal Length (pixels)**	**Principal Point (pixels)**	**Mean Reprojection Error (pixels)**	**SD (pixels)**
A5-1	926.0, 926.0	499.2, 348.6	1.4	4.5
A6-1	857.8, 857.8	255.4, 338.6	1.6	5.3
A6-5	920.5, 920.5	314.0, 240.9	1.3	4.7
A6-6	842.3, 842.3	296.6, 145.4	0.7	4.2
A6-9	747.3, 747.3	366.6, 226.6	8.8	12.4
B5-3	801.2, 801.2	364.1, 202.1	5.3	6.1
B6-1	597.9, 597.9	388.8, 219.8	0.9	1.6
B6-2	817.9, 817.9	295.1, 262.1	2.6	4.4
B6-3	804.2, 804.2	374.1, 246.1	4.7	7.5
B6-4	824.1, 824.1	336.6, 237.2	3.6	6.5
B6-5	840.0, 840.0	317.9, 229.6	2.2	4.3
B6-6	862.5, 862.5	320.1, 247.1	4.6	8.5

**Table 2.** Estimated external camera parameters of the BRL scenario.

**Camera**	**Position (m)**	**Orientation (radians)**	**Ground Truth Position (m)**	**Elapsed Time (s)**
A5-1	−37.93, −25.37, 3.88	−0.63, 1.85, 2.03	−37.91, −28.2, 3.75	95.2
A6-1	42.84, −25.47, 4.06	1.57, −0.73, −1.37	42.66, −24.95, 3.99	86.5
A6-5	12.76, −28.10, 2.58	1.61, −0.90, −1.18	12.39, −28.95, 3.10	45.2
A6-6	9.83, −22.91, 3.07	0.70, −2.21, −2.10	9.44, −23.5, 2.95	79.6
A6-9	19.32, −4.02, 3.49	0.10, 1.85, 1.94	19.03, −6.65, 3.11	56.8
B5-3	−39.93, −1.16, 2.23	1.20, 1.25, 0.88	−38.94, −2.63, 1.95	69.5
B6-1	52.22, 19.14, 3.53	1.43, 0.60, −0.09	51.98, 18.55, 3.11	66
B6-2	51.66, 7.50, 3.66	1.52, 0.45, −0.09	51.55, 7.20, 3.45	107.3
B6-3	50.48, 2.53, 3.10	1.50, −0.60, −1.33	50.18, 4.36, 2.90	98.6
B6-4	34.45, 1.21, 3.13	1.15, 1.17, 0.42	32.85, 1.63, 2.90	76.8
B6-5	31.80, 6.34, 2.93	1.50, −0.94, −1.31	31.88, 7.48, 5.10	123.9
B6-6	31.81, 14.74, 5.82	1.50, −0.82, −1.32	31.64, 15.59, 5.10	103.2

**Table 3.** Estimated internal camera parameters for the FME scenario.

**Camera**	**Focal Length (pixels)**	**Principal Point (pixels)**	**Mean Reprojection Error (pixels)**	**SD (pixels)**
Cam-mean	2989.6, 2998.9	499.2, 348.6	9.8	15.3
Cam-1	2985.2, 2986.7	493.2, 343.2	15.3	26.8
Cam-2	2985.5, 2980.2	490.2, 346.1	1.2	3.6
Cam-3	2985.7, 2983.2	560.2, 350.8	2.4	6.0
Cam-4	2984.3, 2987.4	510.2, 340.3	3.6	9.0
Cam-5	2989.4, 2987.2	493.2, 341.6	6.5	8.6

**Table 4.** Estimated external camera parameters for the FME scenario.

**Camera**	**Position (m)**	**Orientation (radians)**	**Ground Truth Position (m)**	**Elapsed Time (s)**
Cam-1	−21.29, 3.39	−1.21, −0.90, −1.76	−21.73, 4.04	80.9
Cam-2	0.04, −13.42	0.45, −2.23, 0.37	−0.02, −12.75	106.1
Cam-3	43.83, 15.27	2.27, 0.18, 2.13	42.70, 15.80	182.1
Cam-4	−8.61, −4.81	1.40, −1.27, 1.25	−9.38, −5.73	95.6
Cam-5	−13.97, −0.44	−0.81, −0.80, −1.77	−14.14, −0.56	112.7
